# Supplementary material for: A striatal interneuron circuit for continuous target pursuit
Source: Nat Commun. 2019 Jun 20;10:2715. doi: 10.1038/s41467-019-10716-w (PMC6586681; doi:10.1038/s41467-019-10716-w)
Supplement: Supplementary file 1 — Supplementary Information [file 41467_2019_10716_MOESM1_ESM.pdf]

Supplementary information

A striatal interneuron circuit for continuous target pursuit

Kim et al.

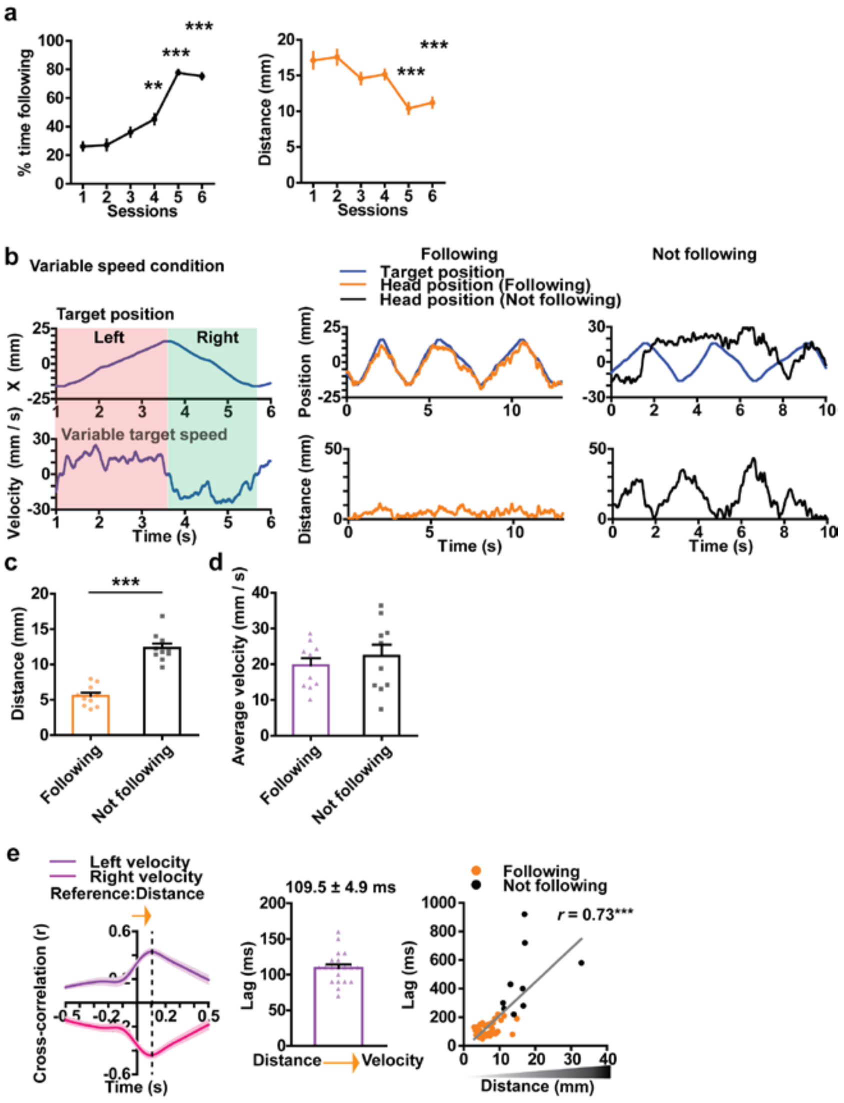

Supplementary Fig. 1. Behavior summary

a) In 6 mice, we recorded behavior with 3D motion capture during acquisition of the continuous pursuit task. Behavioral performance improved in the course of training. Time spent following the target increased significantly (Repeated measures ANOVA, main effect of sessions, $F_{5, 35} = 59.10$, $p < 0.0001$). Distance error is reduced in the same period (Repeated measures ANOVA, main effect of sessions, $F_{5, 35} = 16.76$, $p < 0.0001$).
b) Representative traces of mouse head and target position during a session with variable target velocity. Left panel shows the head and target positions during 'Following.' Right panel shows the head and target positions during 'Not following' in which two positions are different.
c) Self-target distance error is significantly lower during following than not following (paired t-test, $p < 0.0001$). Error bars indicate ± s.e.m. *** $p < 0.0001$.
d) Average velocity is similar between following and not following ($p = 0.053$).
e) Illustration of the temporal relationship between distance and self-velocity. Cross correlation analysis reveals the lag between these two variables, showing that distance

leads self-velocity during pursuit behavior. In addition, the lag shows a positive correlation with distance (r = 0.73, p < 0.0001). When the mouse is actively pursuing and maintaining a small distance, the lag between distance and velocity is small, but the lag increases as pursuit performance declines. Dashed line indicates the time of peak positive and negative correlation. Shaded areas indicate ± s.e.m. Source data are provided as a Source Data file.

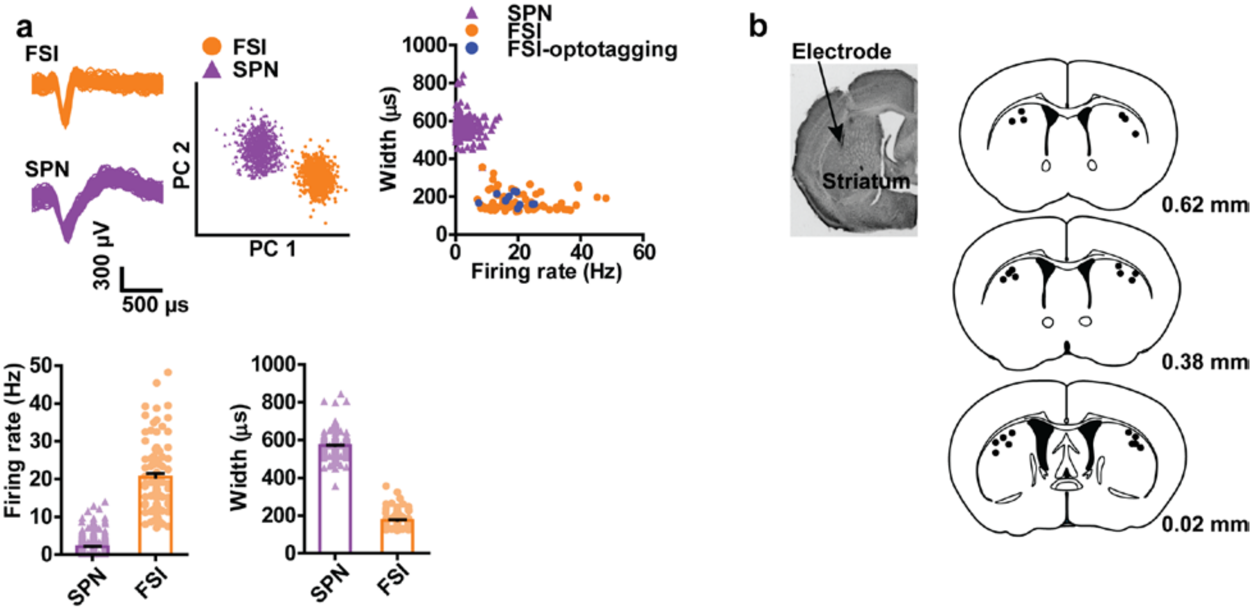

Supplementary Fig. 2. Summary of in vivo electrophysiology

a) SPNs and FSIs are classified based on their spike waveforms and firing rates.
b) Placement of electrodes in the sensorimotor striatum. Source data are provided as a Source Data file.

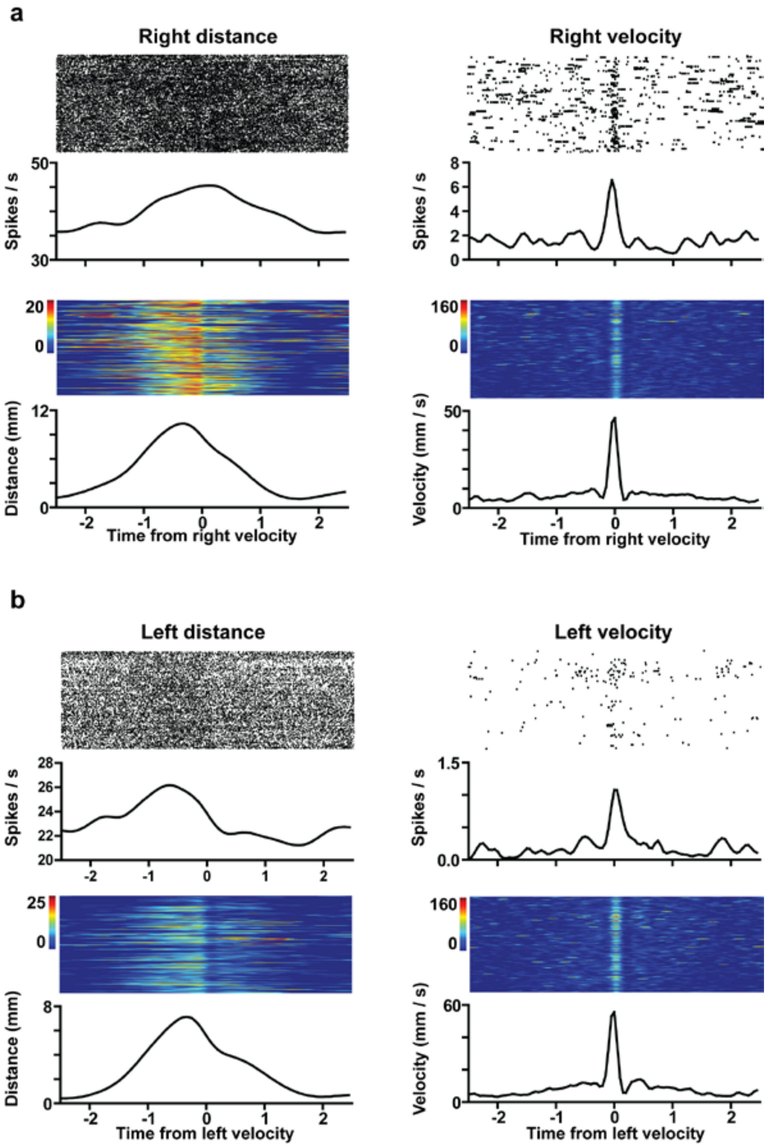

Supplementary Fig. 3. Distance and velocity classification

a) A representative examples of right distance FSI and right velocity SPN from the same animal aligned by right velocity. It is clear from these examples that, while distance and velocity co-vary, these two variables can also be dissociated. FSIs, which show typical tonic activity, are suitable for representing distance, whereas SPNs, which show sparse firing, are suitable for representing velocity.

b) A representative examples of left distance FSI and left velocity SPN from the same animal aligned by left velocity.

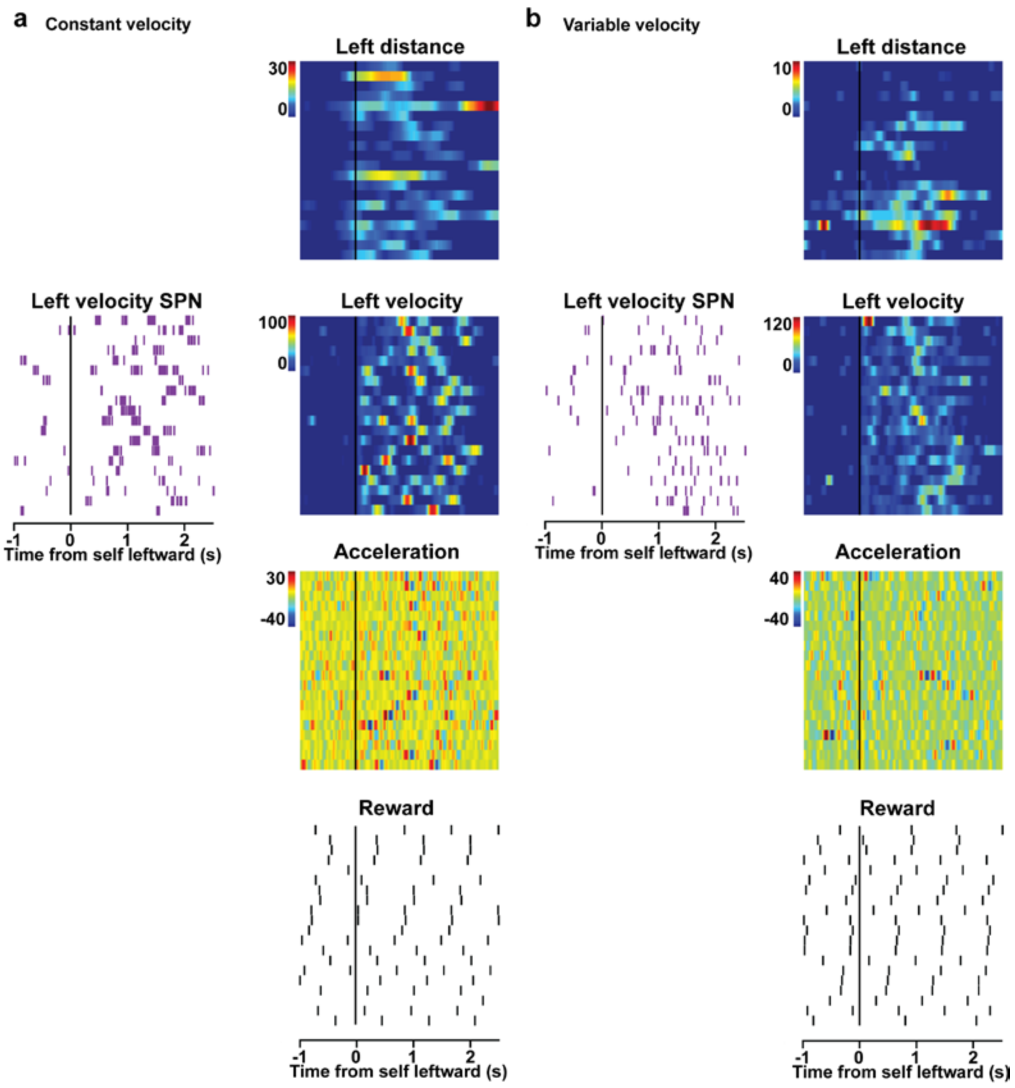

Supplementary Fig. 4. Representative velocity-correlated SPN

a) In constant target velocity condition, correlation between a representative left velocity SPN and different behavioral variables (distance in mm, velocity in mm/s, acceleration in mm/s^2, and reward in sucrose drops, 20 consecutive target movements while the animal is following).
b) In variable target velocity condition, correlation between a representative left velocity SPN and different behavioral variables.

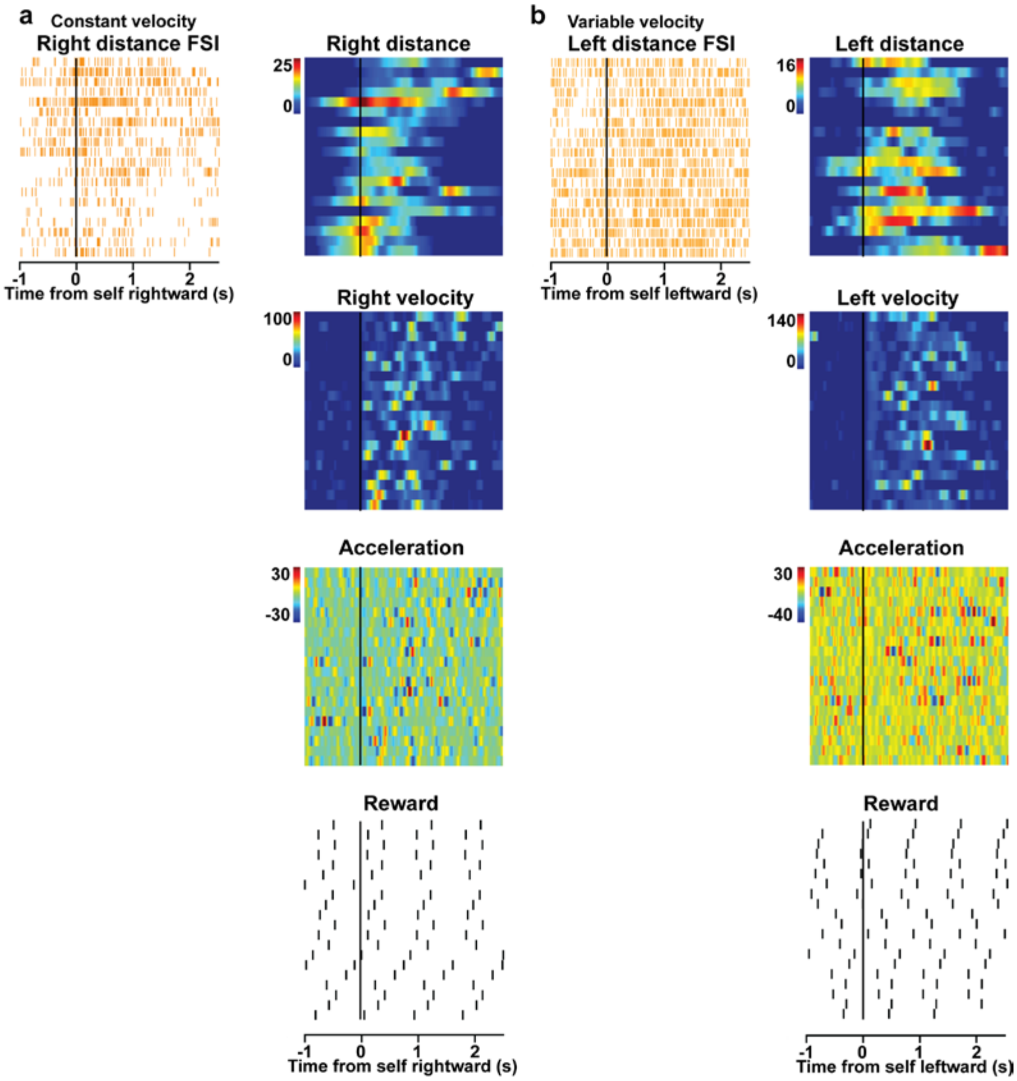

Supplementary Fig. 5. Representative distance-correlated FSI

a) In constant target velocity condition, correlation between a representative right distance FSI and different behavioral variables ((distance in mm, velocity in mm/s, acceleration in mm/s^2, and reward in sucrose drops, 20 consecutive target movements while the animal is following).
b) In variable target velocity condition, correlation between a representative Left distance FSI and different behavioral variables.

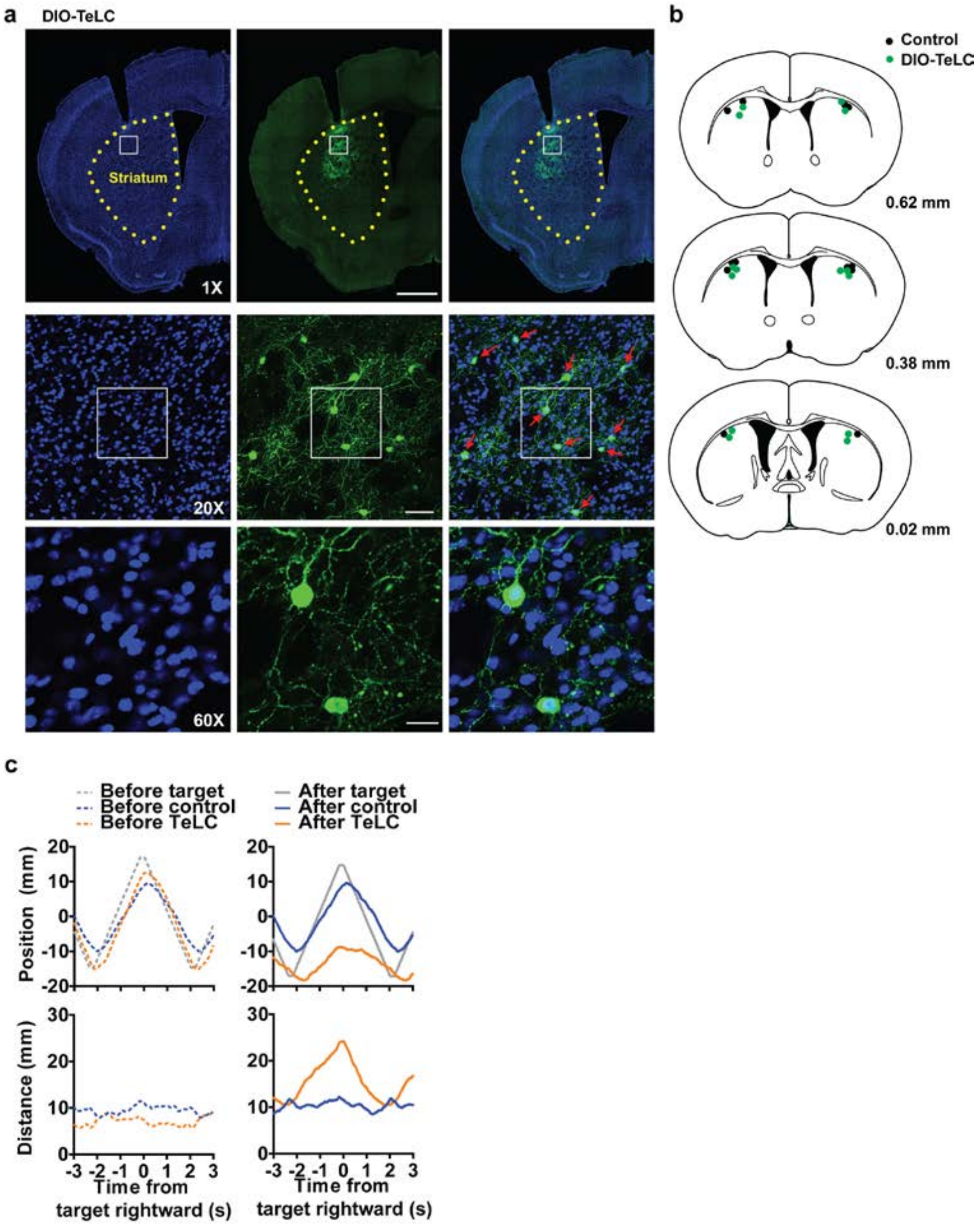

Supplementary Fig. 6. Summary of TeLC expression.

a) Coronal section showing representative expression of GFP-tagged TeLC in the striatum. Left, DAPI staining. Middle, GFP staining. Right, merged.
b) Locations of TeLC injection regions in the striatum.
c) A representative example of target and head positions before and after TeLC injections (top). Distance error was also increased by TeLC (bottom). Both groups showed successful following behavior before TeLC injections, but silencing of FSI activity impaired pursuit performance, causing increased distance error.

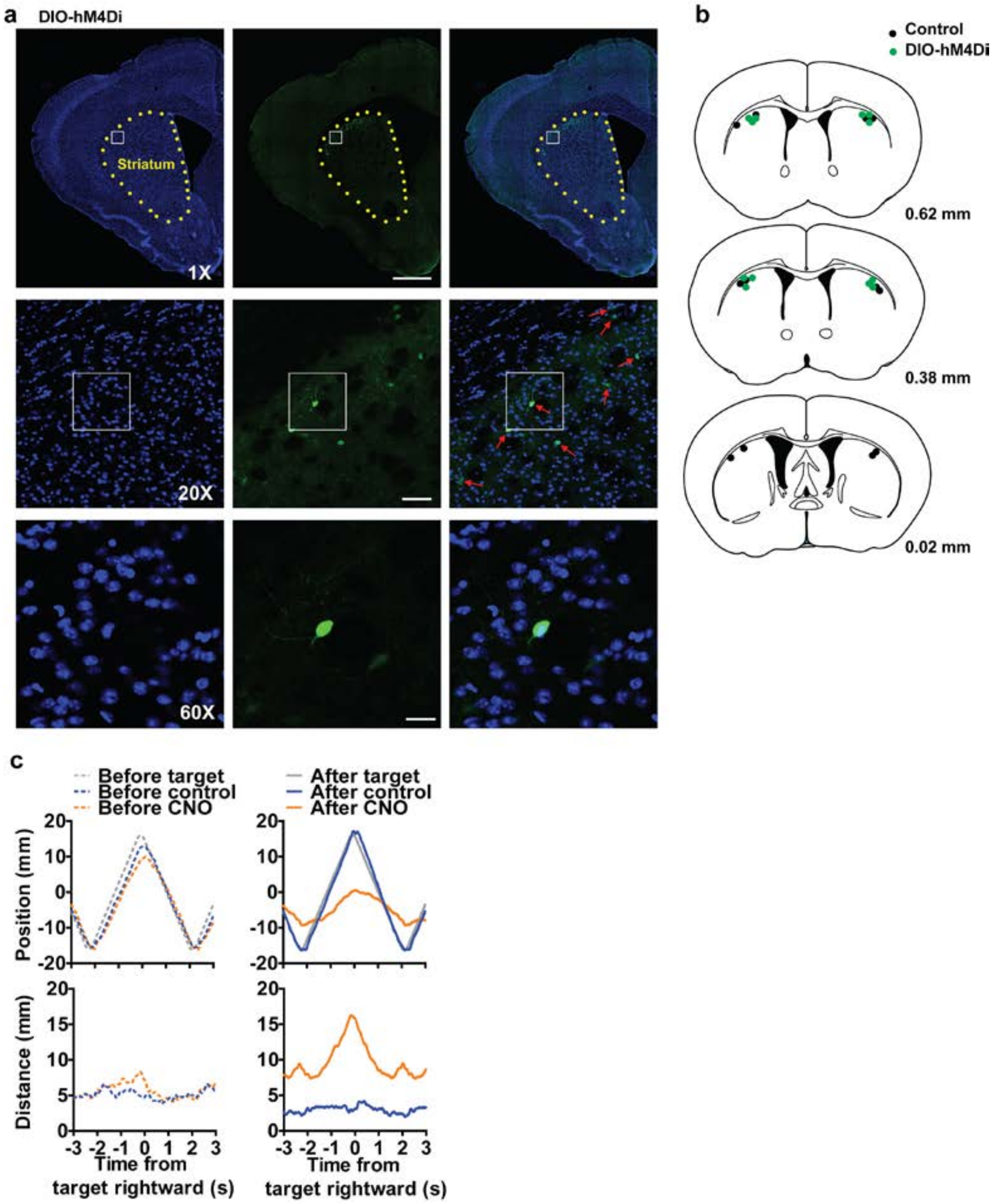

Supplementary Fig 7. Summary of hM4Di expression

a) Coronal section showing representative expression of GFP-tagged hM4Di in the striatum. Left, DAPI staining. Middle, GFP staining. Right, merged.
b) Locations of hM4Di injection regions.
c) A representative example of target and head positions (control: blue; CNO hM4Di: orange) before and after CNO injection (top). Distance error was also increased by hM4Di (bottom). Both groups showed successful following behavior before CNO injections. However, blockade of FSI activity by CNO injection showed poor following behavior, causing increased distance.

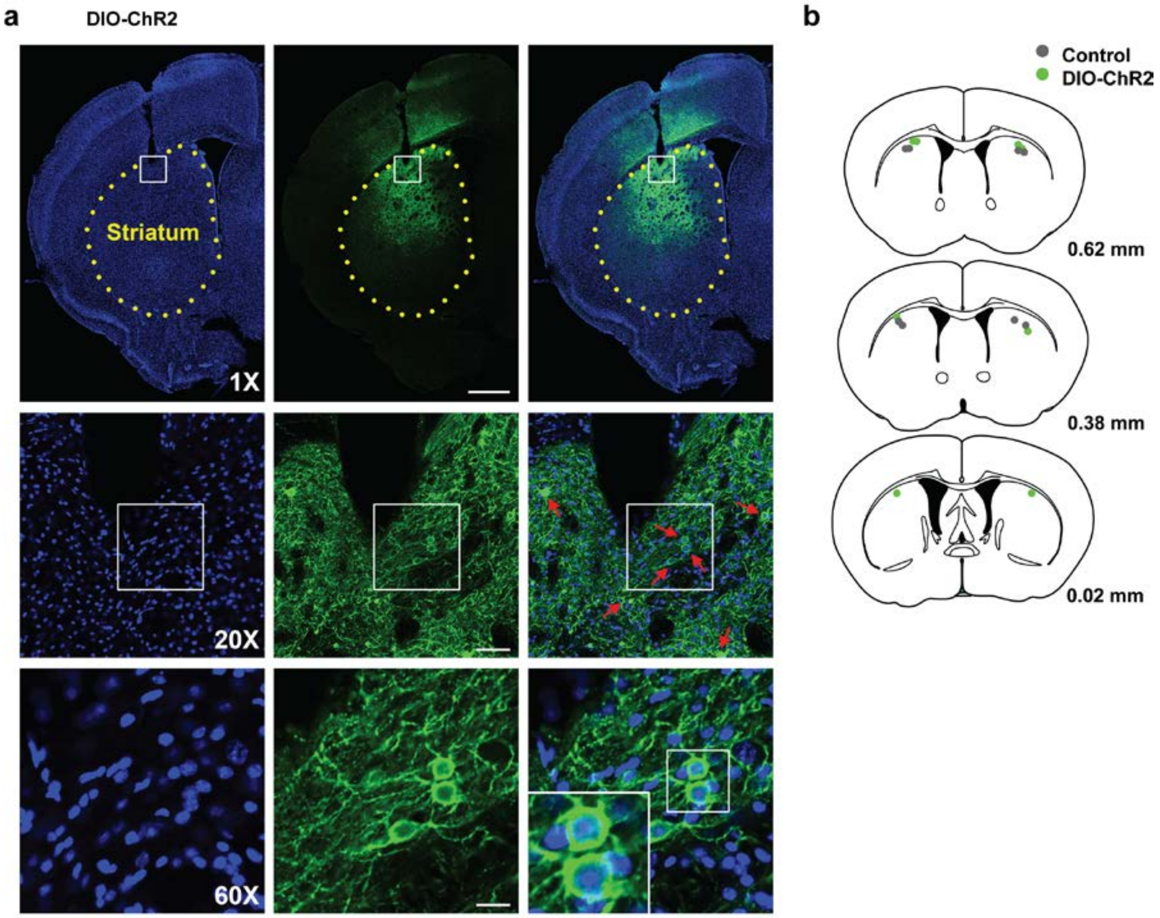

Supplementary Fig. 8. Summary of ChR2 expression

a) Coronal section showing expression of DIO-ChR2 in the sensorimotor striatum. Left, DAPI staining. Middle, GFP staining. Right, merged.
b) Locations of optic fibers.

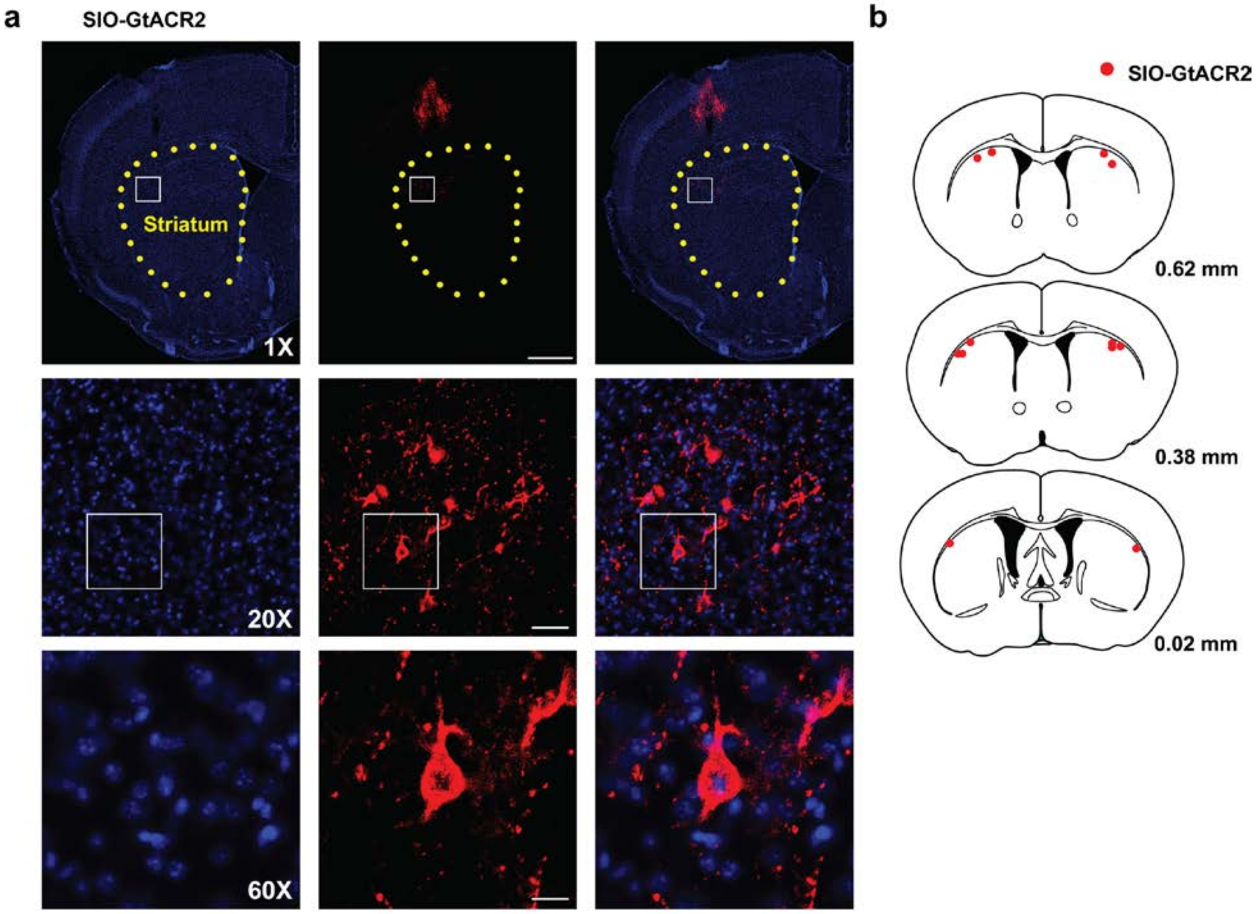

Supplementary Fig. 9. Summary of SIO-GtACR2 expression

a) Coronal section showing expression of FusionRed-tagged GtACR2 in the sensorimotor striatum. Left, DAPI staining. Middle, FusionRed staining. Right, merged.
b) Locations of optic fibers.

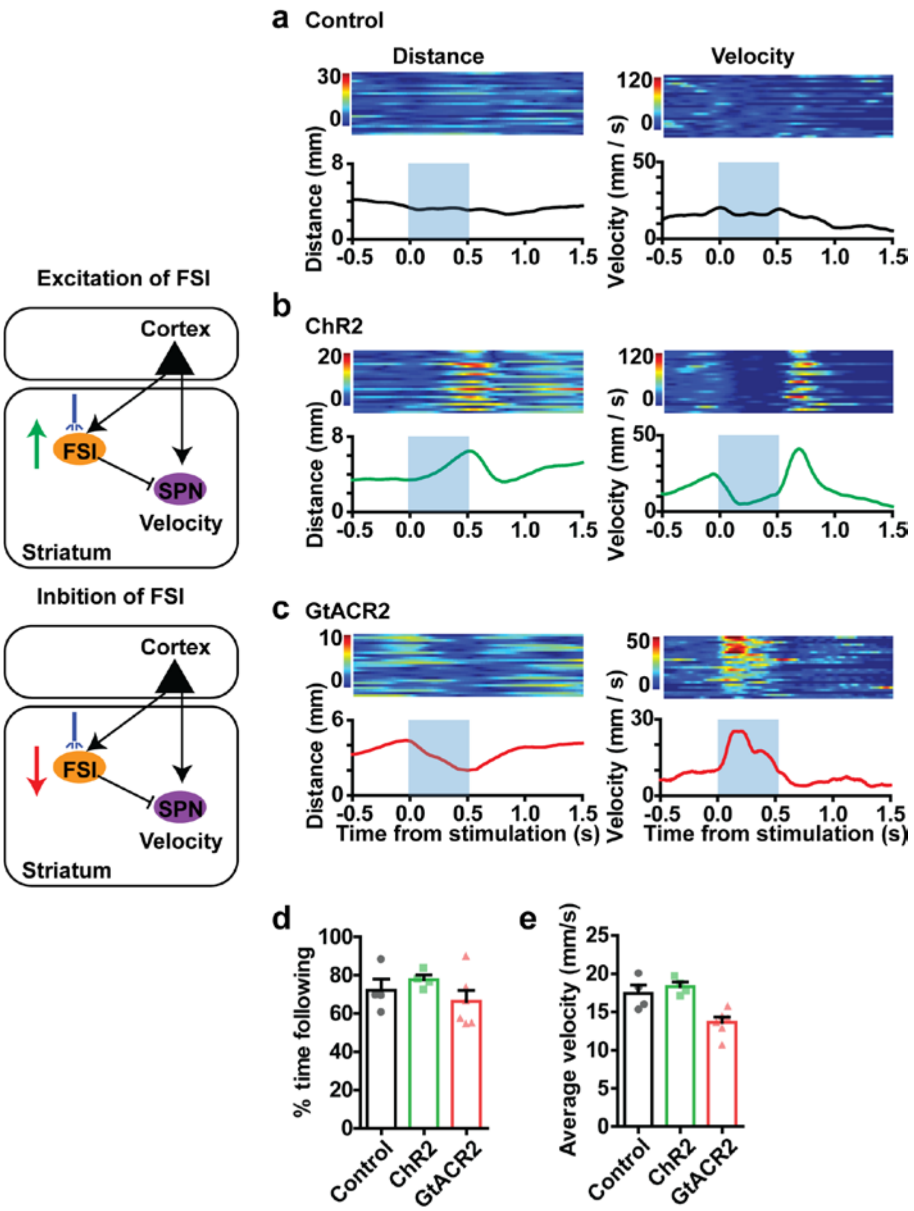

Supplementary Fig 10. Summary of optogenetic manipulation of FSIs.

a) Control group showed no change in distance or velocity.
b) DIO-ChR2 group showed decreased velocity and increased distance during photo-stimulation.
c) SIO-GtACR2 group showed increased velocity and decreased distance during photo-stimulation.
d) Overall % time spent following is not altered by stimulation.
e) SIO-GtACR2 group showed slightly decreased overall average velocity ($p < 0.05$). Source data are provided as a Source Data file.

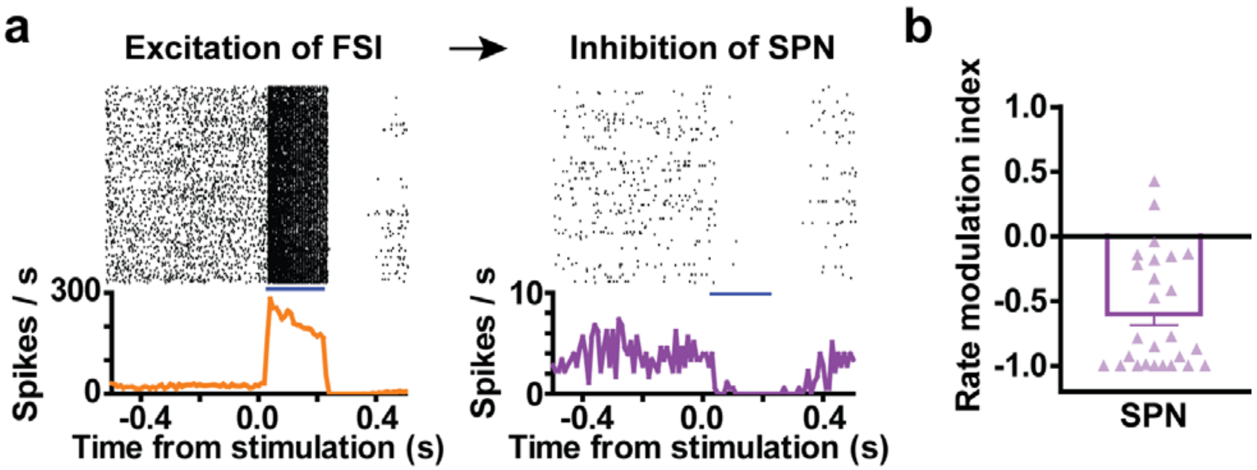

Supplementary Fig. 11. FSI-SPN interaction

a) Excitation of FSI with ChR2 *in vivo*. In the same mouse, there is disinhibition of SPNs, as shown on the right.
b) Rate modulation index ((Firing $rate_{after}$ – Firing $rate_{before}$) / (Firing $rate_{after}$ + Firing $rate_{before}$)) indicates suppression of SPN activity by excitation of FSIs. Source data are provided as a Source Data file.

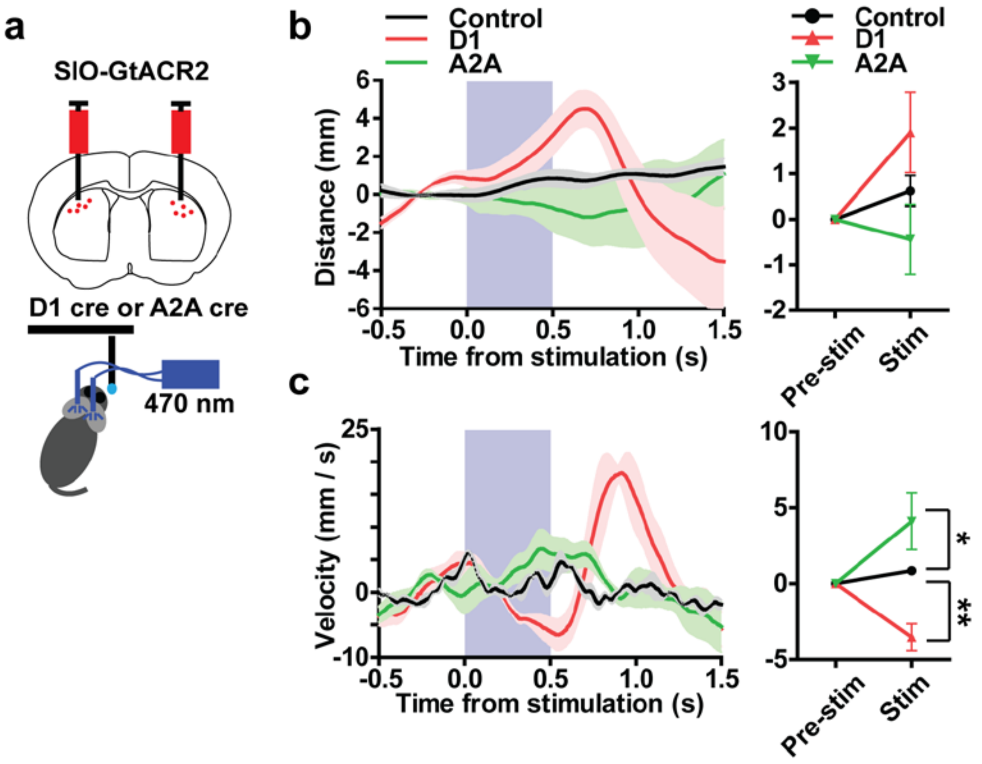

Supplementary Fig. 12. Optogenetic inhibition of SPN activity.

a) We injected Cre-dependent inhibitory channelrhodopsin (AAV-hsyn1-SIO-GtACR2-Fusion Red) to reduce SPN activity in either the direct pathway (D1+ SPNs) or the indirect pathway (A2A+ SPNs).

b) Effect of photo-stimulation on self-target distance. Inhibition of direct pathway or indirect pathway activity did not have a significant effect on distance (repeated measures two-way ANOVA, Interaction: $F_{2, 7} = 2.484$, $p = 0.1530$, Group: $F_{2, 7} = 2.484$, $p = 0.1530$; Time: $F_{1, 7} = 2.447$, $p = 0.1617$). Control (PV-Cre light only): n = 3 ; D1 SIO-GtACR2: n = 4; A2A SIO-GtACR2: n = 3. Error bar indicates ± s.e.m.

c) Effect of photo-stimulation on self velocity. Inhibition of direct pathway activity decreased velocity, whereas inhibition of indirect pathway activity increased velocity (repeated measures two-way ANOVA, Interaction: $F_{2, 7} = 11.82$, $p = 0.0057$, Group: $F_{2, 7} = 11.82$, $p = 0.0057$; Time: $F_{1, 7} = 0.5037$, $p = 0.5008$, control vs D1 SIO-GtACR2, $p < 0.01$; control vs. A2A SIO-GtACR2, $p < 0.05$,). Error bar indicates ± s.e.m. ** $P < 0.01$. *$p < 0.05$. Source data are provided as a Source Data file.

Supplementary Table 1. SPN electrophysiology summary

	Distance (n)	Velocity (n)	Acceleration (n)	Reward (n)	Unclassified (n)	Total (n)
# neurons	5.2% (19)	32.0% (116)	3.3% (12)	0.0% (0)	59.4% (215)	100.0% (362)

Supplementary Table 2. SPN distribution

	Left striatum (n)	Right striatum (n)
Left distance	2.3 % (5)	1.4 % (2)
Right distance	3.2 % (7)	3.6 % (5)
Left velocity	19.8 % (44)	10.7 % (15)
Right velocity	11.3 % (25)	22.9 % (32)
Left acceleration	1.4 % (3)	1.4 % (2)
Right acceleration	2.3 % (5)	1.4 % (2)
Reward	0.0% (0)	0.0% (0)
Unclassified	59.9 % (133)	58.6 % (82)
Total	100.0 (222)	100.0 (140)

Supplementary Table 3. FSI electrophysiology summary

	Distance (n)	Velocity (n)	Acceleration (n)	Reward (n)	Unclassified (n)	Total (n)
# neurons	56 % (51)	11.0 % (10)	5.5 % (5)	0.0% (0)	27.5 % (25)	100.0% (91)

Supplementary Table 4. FSI distribution

	Left striatum (n)	Right striatum (n)
Left distance	22.9 % (11)	20.9 % (9)
Right distance	20.8 % (10)	48.8 % (21)
Left velocity	10.4 % (5)	4.7 % (2)
Right velocity	2.1 % (1)	4.7 % (2)
Left acceleration	2.1 % (1)	0.0 % (0)
Right acceleration	6.3 % (3)	2.3 % (1)
Reward	0.0 % (0)	0.0 (0)
Unclassified	35.4 % (17)	18.6 % (8)
Total	100.0 (48)	100.0 (43)

Supplementary Table 5. FSI optotagging summary

	Distance (n)	Velocity (n)	Acceleration (n)	Reward (n)	Unclassified (n)	Total (n)
# neurons	73.3 % (11)	6.7 % (1)	0.0 % (0)	0.0 % (0)	20.0 % (3)	100.0% (15)

Supplementary Table 6. Calcium imaging summary

	Distance (n)	Velocity (n)	Acceleration (n)	Reward (n)	Unclassified (n)	Total (n)
# neurons	51.4% (19)	18.9% (7)	0.0% (0)	0.0% (0)	29.7% (11)	100.0% (37)

Supplementary Table 7. Distribution of FSIs from calcium imaging

	Left striatum (n)	Right striatum (n)
Left distance	25.9% (7)	80.0% (8)
Right distance	14.8% (4)	0.0% (0)
Left velocity	25.9% (7)	0.0% (0)
Right velocity	0.0% (0)	0.0% (0)
Left acceleration	0.0% (0)	0.0% (0)
Right acceleration	0.0% (0)	0.0% (0)
Reward	0.0% (0)	0.0% (0)
Unclassified	33.3% (9)	20.0% (2)
Total	100.0% (27)	100.0% (10)
