# Supplementary material for: A striatal interneuron circuit for continuous target pursuit
Source: Nat Commun. 2019 Jun 20;10:2715. doi: 10.1038/s41467-019-10716-w (PMC6586681; doi:10.1038/s41467-019-10716-w)
Supplement: Supplementary file 2 — Description of Additional Supplementary Files [file 41467_2019_10716_MOESM2_ESM.pdf]

Description of Additional Supplementary Files

File Name: Supplementary Movie 1
Description: Representative 3D motion capture of a mouse during pursuit behavior (following).

File Name: Supplementary Movie 2
Description: Representative 3D motion capture of a mouse that is not following the target (same mouse as in Supplementary Movie 1).

File Name: Supplementary Movie 3
Description: Representative calcium imaging signals using the UCLA Miniscope.
