# Supplementary figures and images for: A striatal interneuron circuit for continuous target pursuit
Source: Nat Commun. 2019 Jun 20;10:2715. doi: 10.1038/s41467-019-10716-w (PMC6586681; doi:10.1038/s41467-019-10716-w)

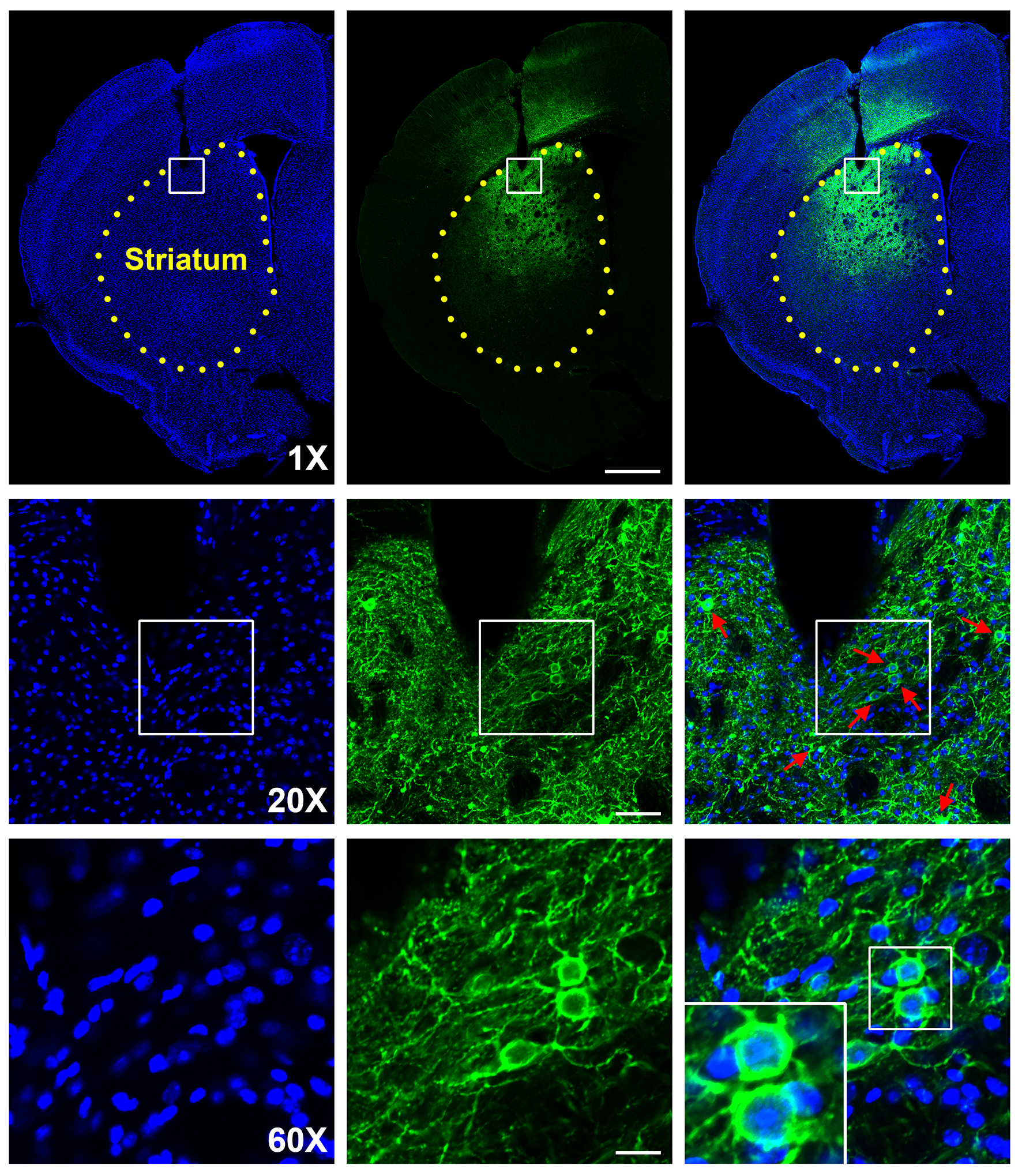

Supplement: Supplementary file 7 — Source Data [file 41467_2019_10716_MOESM7_ESM.zip › E8_ChR2 staining.jpg]

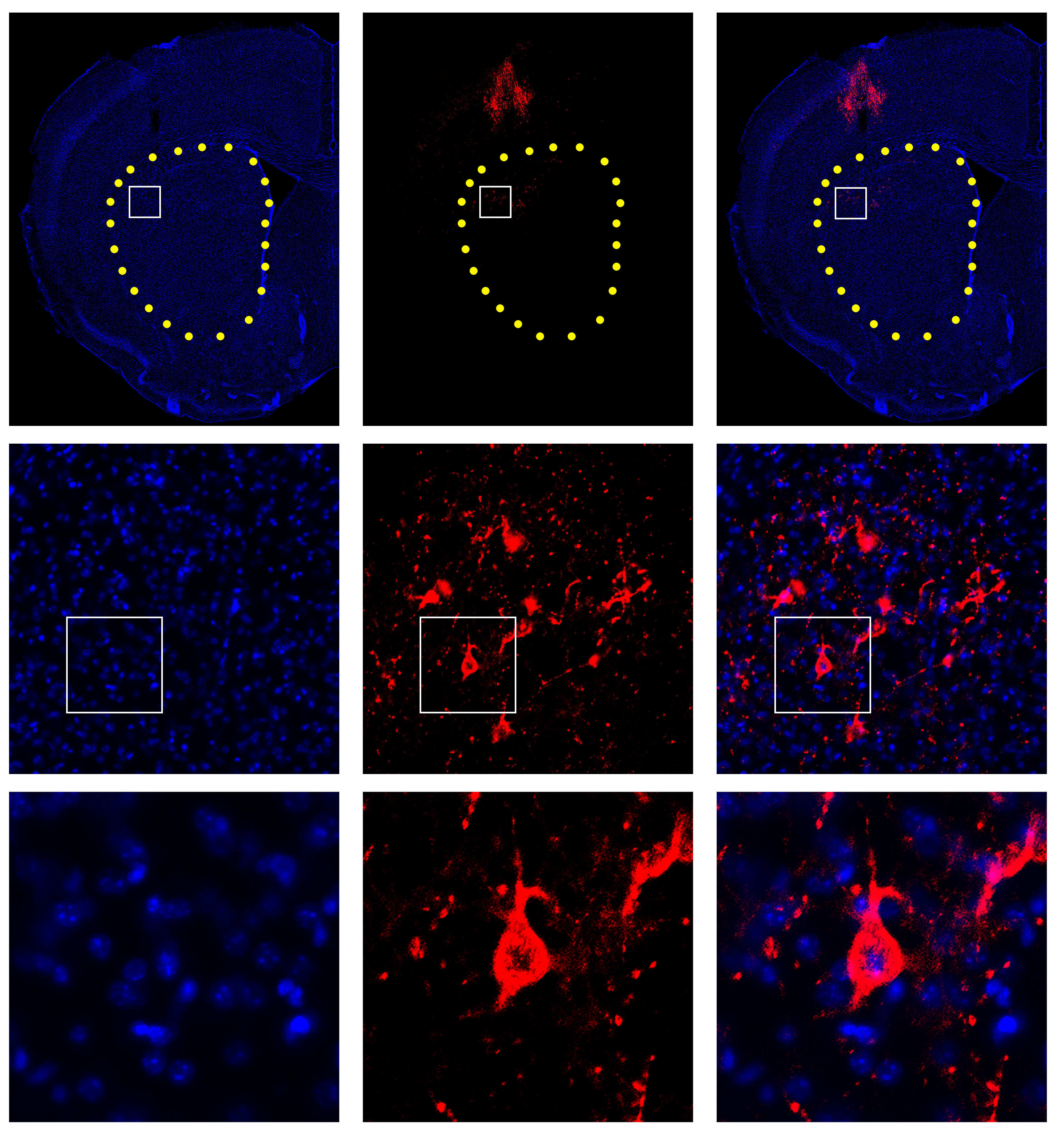

Supplement: Supplementary file 7 — Source Data [file 41467_2019_10716_MOESM7_ESM.zip › E9_GtACR2 staining.jpg]

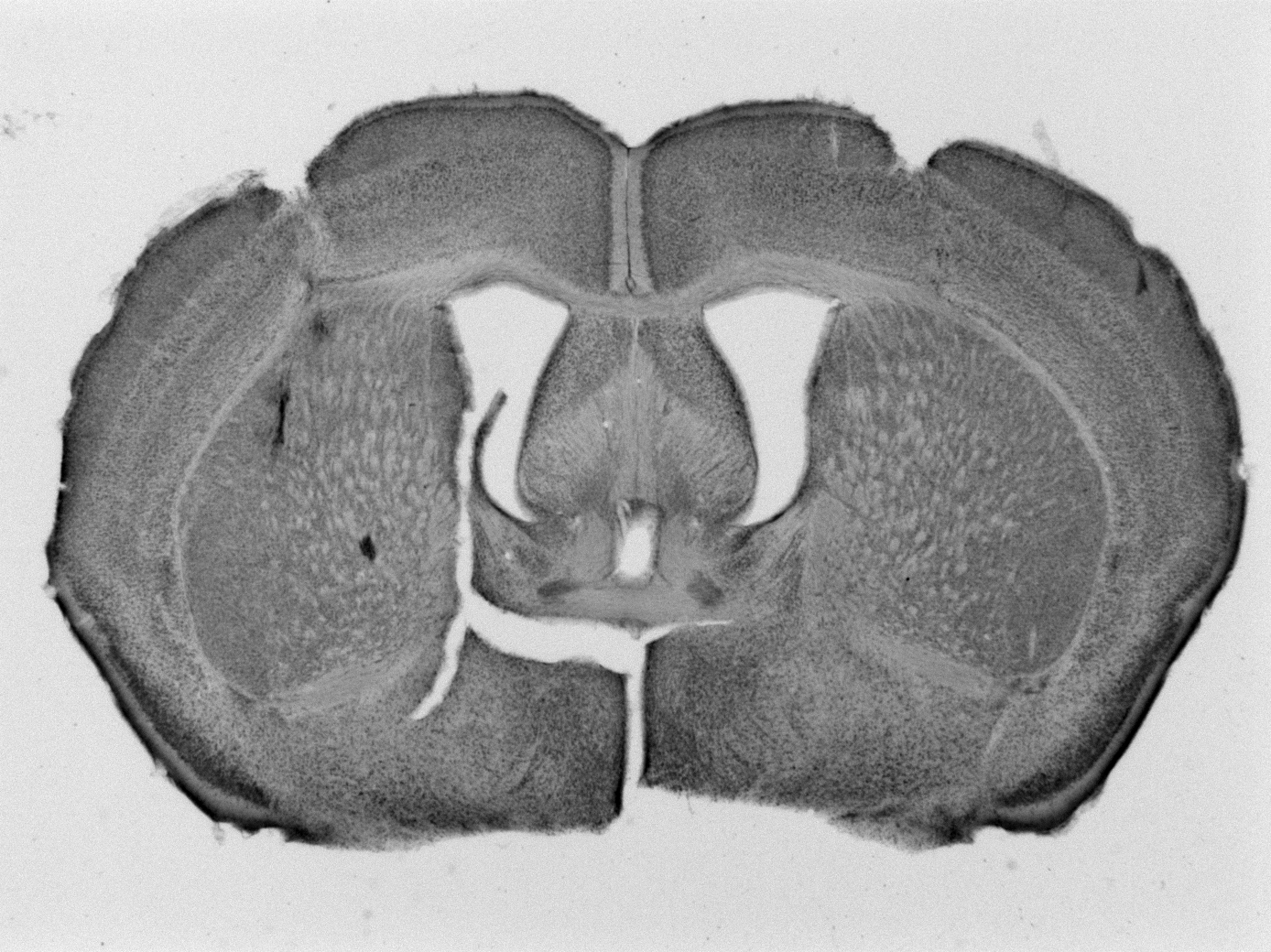

Supplement: Supplementary file 7 — Source Data [file 41467_2019_10716_MOESM7_ESM.zip › E2_Electrode location.tif]

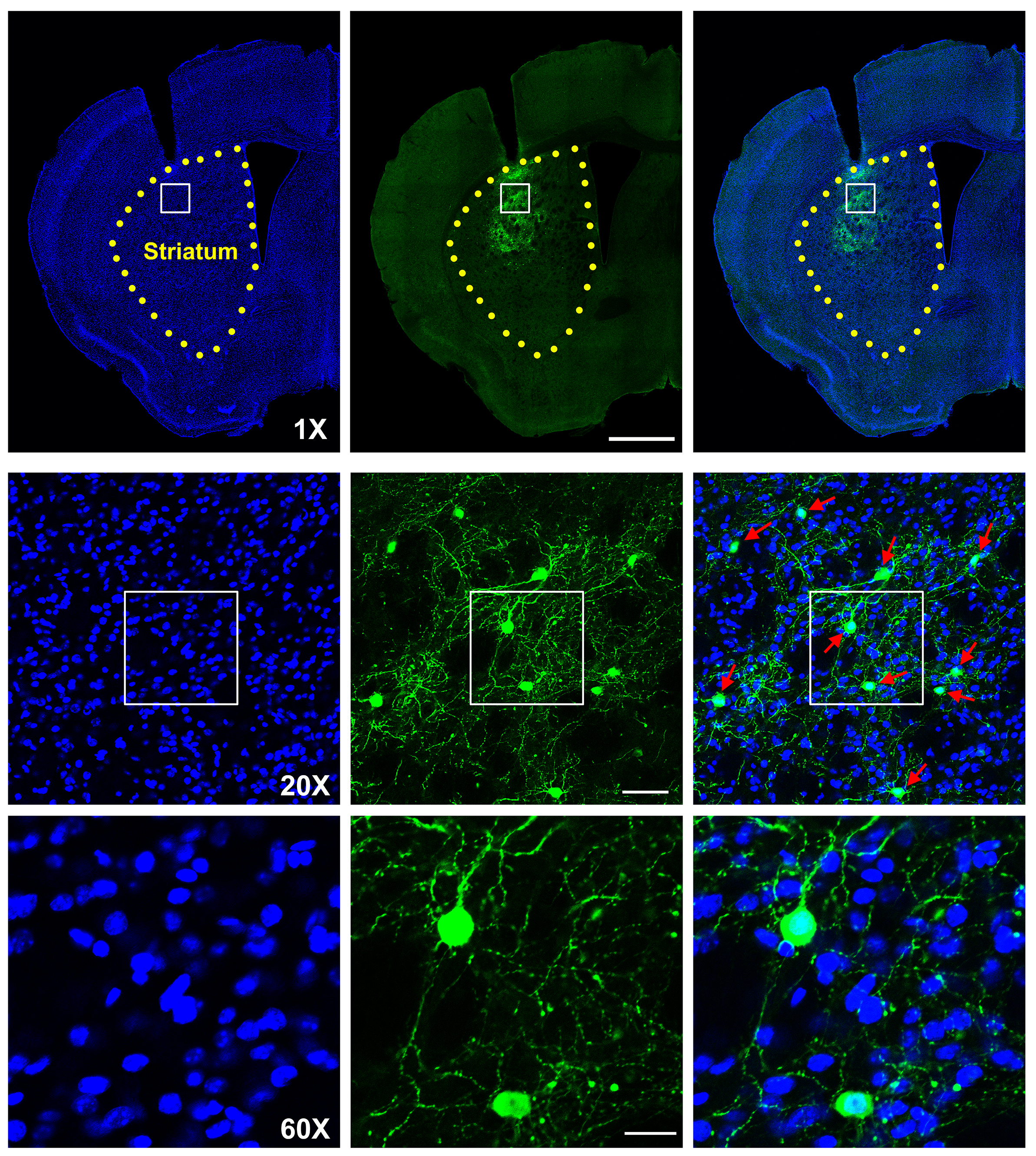

Supplement: Supplementary file 7 — Source Data [file 41467_2019_10716_MOESM7_ESM.zip › E6_TeLC staining.jpg]

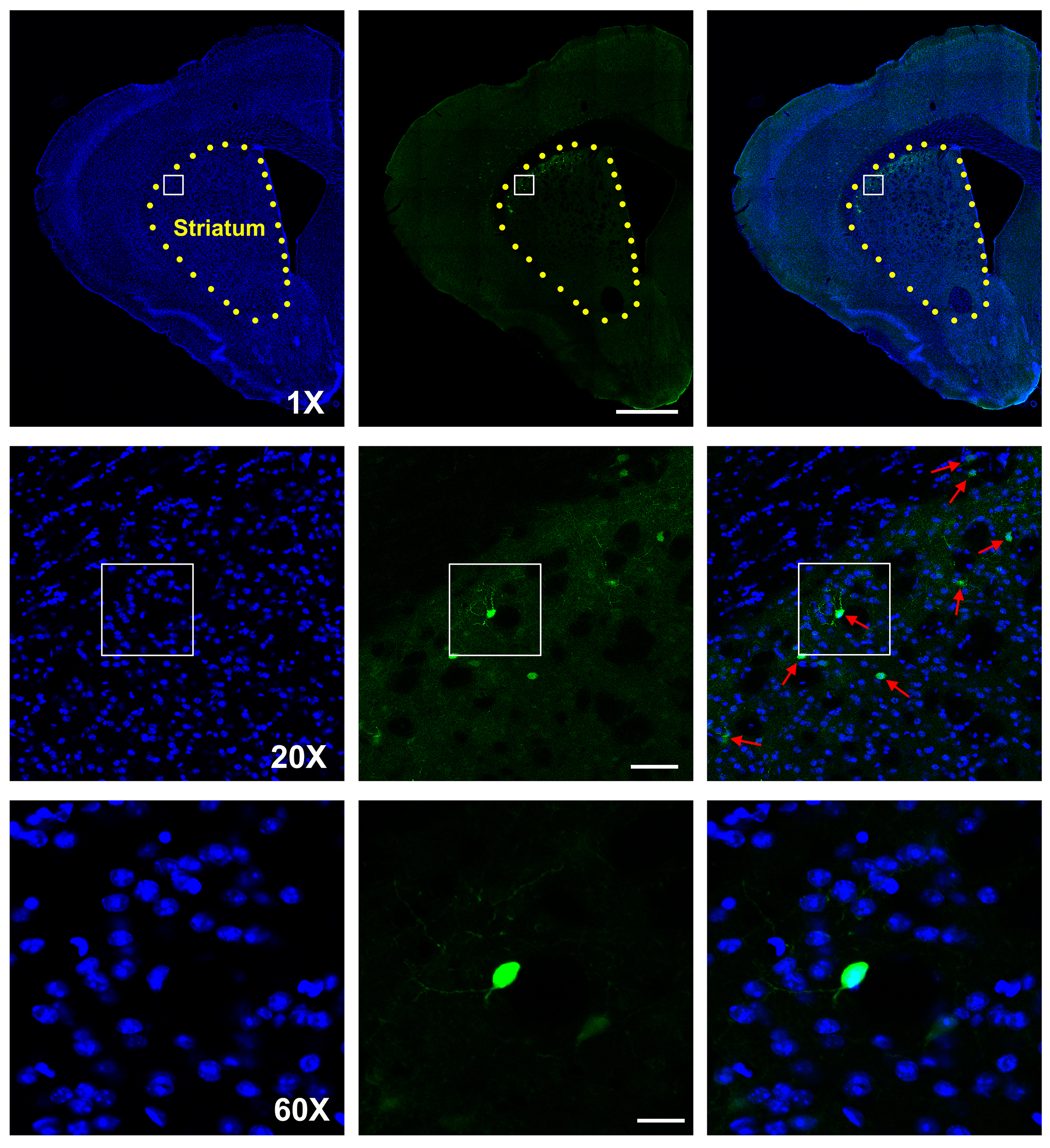

Supplement: Supplementary file 7 — Source Data [file 41467_2019_10716_MOESM7_ESM.zip › E7_DREADD staining.jpg]
